# Teachers’ emotional experiences and exhaustion as predictors of emotional labor in the classroom: an experience sampling study

**DOI:** 10.3389/fpsyg.2014.01442

**Published:** 2014-12-11

**Authors:** Melanie M. Keller, Mei-Lin Chang, Eva S. Becker, Thomas Goetz, Anne C. Frenzel

**Affiliations:** ^1^Department of Empirical Educational Research, University of KonstanzKonstanz, Germany; ^2^Department of Empirical Educational Research, Thurgau University of Teacher EducationKreuzlingen, Switzerland; ^3^Secondary and Middle Grades Education, Kennesaw State UniversityAtlanta, GA, USA; ^4^Department of Psychology, University of MunichMunich, Germany

**Keywords:** teacher emotions, teacher emotional labor, teacher emotional exhaustion, experience sampling method, intra-individual vs. inter-individual analyses

## Abstract

Emotional exhaustion (EE) is the core component in the study of teacher burnout, with significant impact on teachers’ professional lives. Yet, its relation to teachers’ emotional experiences and emotional labor (EL) during instruction remains unclear. Thirty-nine German secondary teachers were surveyed about their EE (trait), and via the experience sampling method on their momentary (state; *N* = 794) emotional experiences (enjoyment, anxiety, anger) and momentary EL (suppression, faking). Teachers reported that in 99 and 39% of all lessons, they experienced enjoyment and anger, respectively, whereas they experienced anxiety less frequently. Teachers reported suppressing or faking their emotions during roughly a third of all lessons. Furthermore, EE was reflected in teachers’ decreased experiences of enjoyment and increased experiences of anger. On an intra-individual level, all three emotions predict EL, whereas on an inter-individual level, only anger evokes EL. Explained variances in EL (within: 39%, between: 67%) stress the relevance of emotions in teaching and within the context of teacher burnout. Beyond implying the importance of reducing anger, our findings suggest the potential of enjoyment lessening EL and thereby reducing teacher burnout.

## INTRODUCTION

It has been recognized that being a teacher is a demanding and sometimes even exhausting job. High dropout rates and the early retirement of teachers (see Macdonald, 1999) have caused some societal alarm in recent years, prompting studies focusing on teacher burnout as a potential cause for teacher attrition (Chang, 2009; see also Ashforth and Lee, 1990). Compared to other professions, teaching in fact poses a relatively high risk of burnout (de Heus and Diekstra, 1999; Brouwers and Tomic, 2000; see also, Maslach et al., 2001). Burnout, defined as “a psychological syndrome in response to chronic interpersonal stressors on the job” (Maslach et al., 2001, p. 399), is conceptualized in scientific studies via three dimensions: emotional exhaustion (EE), depersonalization, and reduced personal accomplishment. EE, considered the core facet of burnout (see for example Maslach et al., 2001; Chang, 2009), refers to having depleted one’s emotional resources and therefore feeling emotionally overextended (Evers et al., 2004). Beyond being related to teachers’ motivation, for example job satisfaction (Wolpin et al., 1991) or enthusiasm (Kunter et al., 2011), EE has also been shown to impact teaching quality (Klusmann et al., 2008). Thus, teacher burnout is a relevant factor in the study of teachers’ professional lives; yet our understanding of the emotional processes in the classroom is still limited (Chang, 2009).

Besides workload and lack of resources, emotional labor (EL) and negative emotions have also been found to be contributing factors in explaining EE (Morris and Feldman, 1996; Abraham, 1999; Chang, 2009, 2013). However, only a few studies have established empirical relationships between these factors in studying teacher emotions (Carson, 2006; Chang, 2009), many of which have relied on cross-sectional and one-time survey data (e.g., Hakanen et al., 2006; Skaalvik and Skaalvik, 2007). In *Emotion in Education*, Schutz and Pekrun (2007) argued for the need to study emotions in real-life contexts and to use multi-method approaches, so that the complexities of emotional processes could be fully understood. In the present study, we examine the links among teacher emotions, EL, and EE with momentary data, utilizing the experience sampling method (ESM).

### RELEVANCE OF TEACHERS’ EMOTIONS TO EMOTIONAL EXHAUSTION

Being a teacher, and teaching in particular, is described as an emotional practice (Hargreaves, 1998), and emotions are characterized as being “an integral part of teachers’ lives” (Sutton and Wheatley, 2003, p. 332). However, scientific studies of teachers’ emotions have only surfaced within the last 15 years. Since then, it has been established that teachers experience a variety of discrete emotions in the course of their professional lives, particularly while delivering instruction (e.g., Nias, 1996; Keller et al., 2014). Emotions are thought to be predictors of teacher behavior in class, in terms of effective instructional practices, as well as student behavior and outcomes (see theoretical model in Frenzel, 2014). Emotions are also relevant within the context of teachers’ health and psychological well-being (for a general discussion, see Fredrickson, 1998).

Outside the teaching profession, there is evidence that burnout – in particular EE – is strongly associated with increased negative affectivity; or conversely, decreased positive affectivity (e.g., Brotheridge and Grandey, 2002). However, in teacher emotion literature, studies addressing this relationship are sparse, even more so when considering discrete teacher emotions in contrast to general affectivity (see Chang, 2009). Kunter et al. (2011) found teacher enthusiasm (regarded as a highly positive affective characteristic of teachers) to be negatively related to burnout (*r* = –0.74 for teaching-related enthusiasm). Similarly, Carson (2006) showed higher levels of teacher burnout corresponded to less positive and increased negative emotions. Chang (2013) investigated teachers’ episodic emotional experiences and how they relate to appraisals, different coping strategies, and ultimately to burnout, finding a clear relationship between teachers’ burnout rates and the increased intensity of negative emotions from disruptive episodes in the classroom. Adding to this evidence, the present study aims to deepen our understanding of the relationship between EE and discrete positive and negative emotional experiences and investigate them on intra- as well as inter-individual levels.

### THE RELATIONSHIP BETWEEN TEACHERS’ EMOTIONAL EXHAUSTION AND EMOTIONAL LABOR

Previous research has identified precursors to teachers’ EE on the class- and school-level, such as student misbehavior (Chang and Davis, 2009) or school climate (Grayson and Alvarez, 2008), as well as on the individual-level, such as self-efficacy (Dicke et al., 2014). Beyond such precursors, EL has been recognized as a central factor involved in the emergence of EE (e.g., Näring et al., 2006; Judge et al., 2009).

Morris and Feldman (1996, p. 987) define EL as “the effort, planning, and control needed to express organizationally desired emotion during interpersonal transactions” and evidence suggests that EL is something teachers report to engage in, on a regular basis (see for example Sutton, 2004; Meyer, 2009) due to the display rules in the classroom. Teachers have implicit rules about whether or not, and when and how to display emotions during instructional time (Sutton, 2004; Schutz et al., 2007), such as the need to show enthusiasm or to remain calm even when class is disrupted. Consequently, teachers feel the urge to regulate their emotions, thereby engaging in EL. The pertinent, albeit dysfunctional, emotion regulation strategy in the context of teacher burnout and particularly regarding EE is surface acting (Näring et al., 2006; Chang, 2013). Surface acting refers to either suppressing the actual yet undesired emotion (e.g., anger), or faking a desired emotion in order to keep up the idealized image (e.g., Brotheridge and Lee, 2003; Hochschild, 2012).

Research shows that the continuous effort of EL is a stressor on teachers that draws on their regulatory resources (Muraven et al., 1998; Muraven and Baumeister, 2000) and causes psychological strain (e.g., Cheung and Tang, 2007; Diestel and Schmidt, 2011). Carson (2007) found surface acting, that is, suppressing, faking, or hiding true emotions, led to greater overall burnout for teachers. Tsouloupas et al. (2010) found direct effects between teachers’ expressive suppression and their EE. More specifically, when teachers reported engaging in expressive suppression, they also reported experiencing increased levels of EE. These results are consistent with those of Brotheridge and Grandey (2002), who found a significant relationship between surface acting (e.g., hiding anger and fear) and EE.

Despite substantial evidence, we still know very little about the relationship between EL and EE when it comes to teachers’ emotional experiences during instruction at the state-level – that is, the level of an individual’s actual experiences at the moment. Yet, teaching and interacting with students is arguably the most important task teachers engage in during the course of their professional lives. Furthermore, display rules primarily stem from beliefs about what is appropriate around *students*; thus, we can assume teachers largely regulate their emotions in class and that emotion regulation plays a subordinate role when preparing lessons at home, for example. Therefore, an investigation is needed that addresses the emotional experiences and EL in the context of teacher burnout, specifically during instructional activities.

### ASSESSMENT OF TEACHERS’ EMOTIONAL PROCESSES

The majority of studies on teacher emotions and EL employs teachers’ generalized self-reports (traits assessed via questionnaires or interviews). However, theoretical considerations (Robinson and Clore, 2002) and empirical investigations indicate that trait-reports on emotional experiences can be biased and do not necessarily reflect an individual’s actual – state-level – experiences (trait-state discrepancy in teachers’ emotion self-reports; see Keller et al., 2014). Also, studies employing teachers’ trait-reports only address inter-individual differences in teachers’ emotional experiences, and not much is known about how the experiences of emotions and EL are related on an intra-individual level. Furthermore, pertinent EL theories are based on intra-individual, that is, situation-specific considerations, such as in a teaching situation when teachers experience an inappropriate emotion, they suppress that emotion, thereby draining their resources. That these relationships also extend to the inter-individual level, that is, between teachers, is implicitly assumed, yet this may not necessarily be the case.

Some research outside of the teaching profession has identified the relationship of state-level emotion labor with EE. Judge et al. (2009) investigated customer service employees’ state-level emotions (i.e., emotions are assessed directly at the moment when they are experienced via the ESM (Scollon et al., 2009). Judge et al. (2009) found the degree to which individuals engaged in surface acting on a daily basis was related to their EE. They concluded that “emotional labor is a dynamic process, wherein the use and consequences of emotional labor vary between-individuals *and* within-individuals” (p. 78).

Carson (2006, Study 2) pioneered the research on teachers’ state-level emotions, assessed directly at the moment they are experienced, and emotion regulation, using ESM. Teachers were asked to report their state-level emotions at different times in a day of teaching (e.g., in the mornings or during mid-day breaks), outside of instructional time in class. Findings indicated that teacher burnout is related to teachers’ emotional experiences, as well as the frequency with which teachers regulate their emotions.

Although much published research focuses on teacher burnout and its diverse antecedents and consequences, little is known about state-level emotional processes (such as emotional experiences and EL) involved in teacher burnout. Even less is known about teachers’ EE based on data drawn from the instructional time or situation, or at an intra-individual level, from lesson to lesson.

## RESEARCH OBJECTIVES AND HYPOTHESES

In response to the notable lack of research addressing teachers’ state-level emotional processes within the context of burnout, this study aims to investigate the relevance of teachers’ state-level emotional experiences (enjoyment, anxiety, and anger), EL, and how the occurrence of emotions and EL in actual classroom situations relates to EE. Particularly, we were initially – on a more exploratory level – interested in determining how pronounced EL gets for teachers during instruction time. This was done by drawing on momentarily assessed EL (state). To our knowledge, ours is the first study that attempts to assess EL and emotional experiences *in vivo*, while teachers are in class. In addition, we investigated how teachers’ trait-reported EE is related to their state reports of emotional experiences. We therefore formulated our first hypothesis:

H1: Emotional exhaustion is negatively related to positive emotions (enjoyment) and positively related to negative emotions (anxiety, anger).

We also expect teachers’ emotional experiences in turn should relate to their EL. We therefore formulated our second hypothesis:

H2: Enjoyment is negatively related to EL, while anxiety and anger are positively related to EL.

Lastly, we investigated how state emotional experiences, trait EE, and trait EL jointly relate to state EL on an intra- and inter-individual level and formulated our third hypothesis:

H3: Emotional exhaustion, trait EL and negative emotions are positively related to state EL, whereas positive emotions are negatively related to state EL.

**FIGURE 1:**
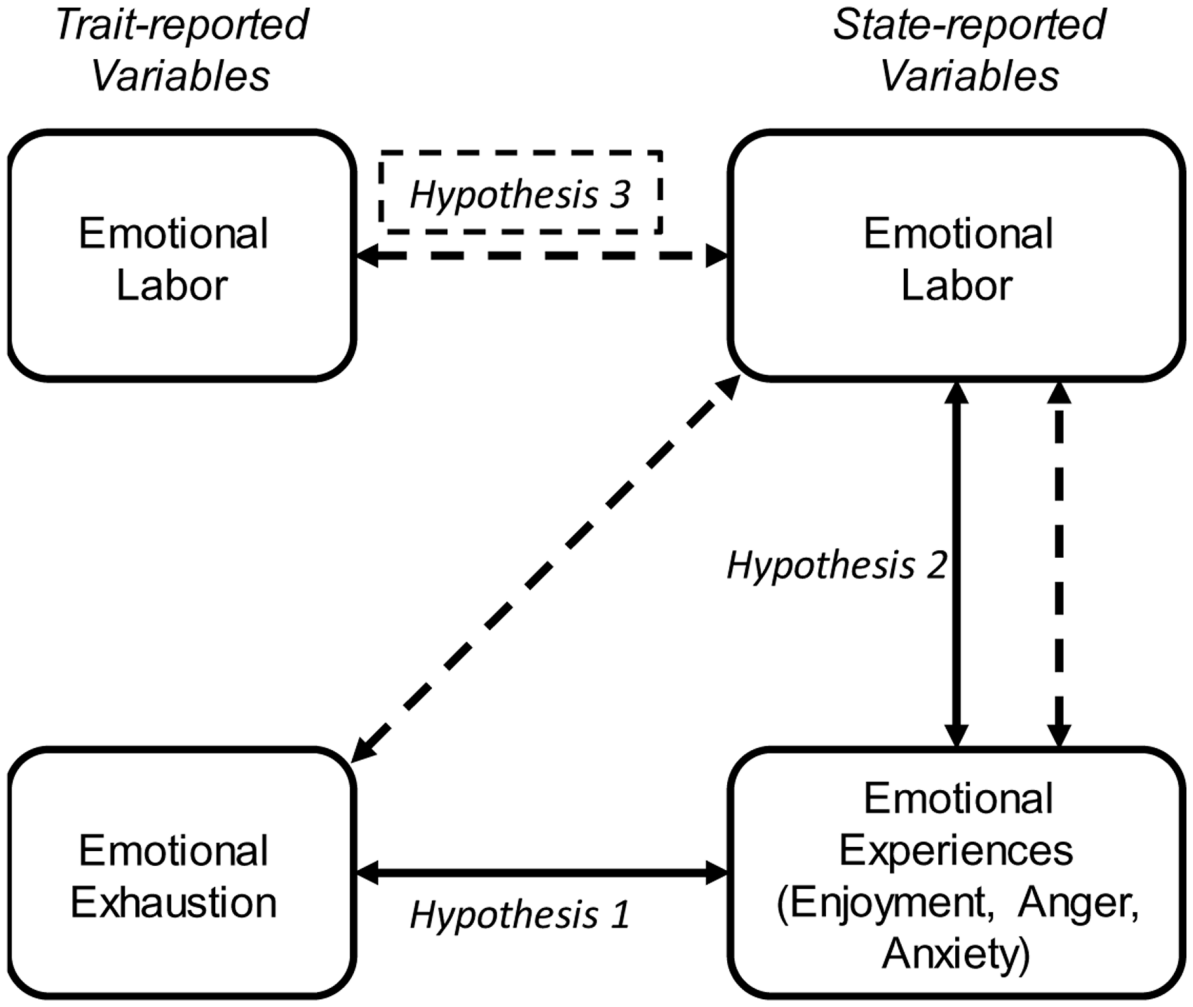
**Figural representation of the study hypotheses.** Relations between state-reported variables were investigated both on an inter- and intraindividual level. The dashed lines represent the testing of Hypothesis 3 in which a multilevel regression model was used to test the influences of trait-reported emotional labor and emotional exhaustion and state-reported emotional experiences on state-reported emotional labor.

The relationships pertaining to these hypotheses are depicted in **Figure 1**.

## MATERIALS AND METHODS

### SAMPLE

The participants in the ESM study were 39 teachers (20 female, 16 male, 3 did not indicate their gender) from the highest track of the German school system, the Gymnasium, which approximately one third of students attend (Federal Statistical Office [Statistisches Bundesamt], 2014). The participants were on average 44.14 years old (*SD* = 11.33 years) and had been teaching for an average of 16.16 years (*SD* = 11.94 years), including the induction phase.

### PROCEDURE

After introducing the research project in participating schools’ staff meetings, appointments were made with all interested teachers so they could obtain more information about procedures and technical issues. The teachers were equipped with paper-and-pencil questionnaires for the trait-based assessment (demographics, trait EL, and EE), and handheld devices (Palm Pilot Z22) for the state-based experience sampling assessment. They were instructed to fill out the trait questionnaire in advance and then they used the handheld device to report state-level data for two consecutive weeks. Teachers were given a demonstration on how to operate the Palm Pilots and were also equipped with a detailed instruction manual. Questionnaires and handheld devices were collected 3–4 weeks later, since teachers did not all start on the same day. The handheld devices were programmed with experience sampling software (PMAT; see Weiss et al., 2004), and data assessment combined event and random sampling. Teachers were instructed to activate the device at the beginning of each regular lesson (i.e., event-sampling). The device was programmed to randomly signal (i.e., random sampling) once within that lesson and presented a short questionnaire. A 5-min response window was programmed into the PDA, so that teachers were not forced to interrupt their lesson in the middle of a sentence. If the teacher did not answer the question set within 5 min after the initial alarm, the PDA automatically ended the question set and saved it as a “missed signal”; this happened for about 10% of signals (mostly due to the signaling noise being too low). Verbal feedback given by the teachers after the ESM-period indicated that teachers were able to implement the ESM with relative ease into their teaching and overall, they did not find it intrusive. Teachers activated their devices in 20 school lessons on average, and it took them approximately half a minute (*M* = 37 s, *SD* = 21 s) to answer the set of questions. Altogether, the experience sampling assessment yielded *N*= 794 state assessments.

### MEASURES

#### Trait-reported emotional exhaustion

Teachers’ EE was assessed using the respective subscale of the Maslach Burnout Inventory (Maslach et al., 1996) which was translated into German by Enzmann and Kleiber (1989). It consists of nine items which were rated on a five-point scale from 1 (*not true at all*) to 5 (*completely true*), with a sample item being “I feel fatigued when I get up in the morning and have to face another day at school.” The scale showed good reliability (Cronbach’s Alpha = 0.87).

#### Trait-reported emotional labor

To assess teachers’ EL, a modified measure of the Frankfurt Emotion Work Scale (Zapf et al., 1999) by Neubach and Schmidt (2006) was utilized. Its five items were adapted to match the target group of teachers (substituting “work” with “class” and “customers” with “students”). A sample item was as follows: “How often do you have to show feelings in class that you do not really feel?” The items were rated on a five-point scale from 1 (*never*) to 5 (*very often*). The scale achieved high reliability (Cronbach’s Alpha = 0.91).

#### State-reported emotional labor

To measure teachers’ state-reported EL, two items from the trait EL scale were adapted to suit the momentary assessment. The items were as follows: “At the moment I have to suppress my feelings” and “At the moment, I have to display emotions that do not correspond to my inner feelings,” both of which could be rated on a five-point scale ranging from 1 (*not true at all*) to 5 (*completely true*). The items represent the two surface acting strategies, namely suppression and faking, and were highly correlated (*r* = 0.63, *p* < 0.001 for within-level and *r* = 0.86, *p* < 0.001 for between-level). Both items were subsequently combined into on overall scale for further analysis.

#### State-reported emotions

To represent teachers’ relevant emotional experiences, we chose the most frequently experienced positive and negative emotions, enjoyment and anger, respectively (Keller et al., 2014). Furthermore, we included anxiety as a particularly detrimental teacher emotion (see Frenzel et al., 2009; Frenzel, 2014). Due to time constraints for the ESM assessment, we relied on single items to assess teachers’ state emotional experiences (for a similar single-item assessment of emotions, see for example, Nett et al., 2011; Goetz et al., 2013). The respective items were formulated as follows: “At the moment, how strongly do you experience enjoyment/anger/anxiety?,” and they could be rated on a five-point scale from 1 (*not at all*) to 5 (*very strongly*).

### STATISTICAL ANALYSES

Our sample represented a nested data structure with measures on level 1 (*N*_1_ = 794) nested within persons on level 2 (*N*_2_ = 39). To correctly estimate the standard errors in such a nested data structure, we conducted multilevel regression analyses (random intercepts and slopes) to test for our research hypotheses, using the M*plus* 7.0 software (Muthén and Muthén, 1998–2012). As such, relationships could be modeled separately for the within- and between-levels.

To test Hypothesis 1, we ran three random intercept models with EE (as a between-level variable) predicting the emotional experiences of enjoyment, anxiety, and anger (as within-level variables). The respective equations for these models were Level 1: Emotion*_ij_* = β_0_*_j_* + *r_ij_*, and Level 2: β_0_*_j_* = γ_00_ + γ_01_ EE*_j_* + *u*_0_*_j_*.

Regarding Hypotheses 2 and 3, we ran three successive random intercept and slope models. In Model 1 (M1), state EL is predicted by emotional experiences both introduced as groupmean-centered Level 1 predictors indicating the within-person effects of emotions on EL, and aggregated grandmean-centered Level 2 predictors indicating the between-person effects of emotions on EL. In Model 2 (M2), EE and trait EL (as between-level variables) predict state EL. Finally, in Model 3 (M3), all predictors are simultaneously included. For this final model, the equations are as follows:

$$Level\,\;\;1:EL_{ij}=\beta_{0j}+\beta_{1j}Enj_{ij}+\beta_{2j}Anx_{ij}+\beta_{3j}Ang_{ij}+r_{ij};$$

$$Level\,\;\;2:\beta_{0j}=\gamma_{00}+\gamma_{01}{Enj}_{j}+\gamma_{02}{Anx}_{j}+\gamma_{03}{Ang}_{j}+\gamma_{04}{EE}_{j}+\gamma_{05}{ELtrait}_{j}+u_{0j};$$

$$\beta_{1\,j}=\gamma_{10}+u_{1\,j};$$

$$\beta_{2\,j}=\gamma_{20}+u_{2\,j};$$

$$\beta_{3\,j}=\gamma_{30}+u_{3\,j}\,.$$

We recognize that the influences of the emotions and emotion-related variables could go both ways in real-life situations. However, given the limits in the methodology we have chosen (administering survey before collecting ESM data), we did not further test for directionality and the reverse effects among these variables. Limitations due to the decisions for the analyses are further discussed in a later section.

### ETHICAL PRINCIPLES

The present study was conducted abiding by the ethical principles provided by the German Psychological Society [DGPs] (2007) and the American Psychological Association [APA] (2010). Guidelines provided by these institutions state that formal informed consent is not necessary when no potential harm or distress is to be expected and/or when normal educational practices are followed as a goal of the research. Prior to their participation, the participants of the present research were informed of the research, duration, and procedures. Participation was voluntary and participants provided verbal informed consent prior to data collection. All data was collected and analyzed anonymously.

## RESULTS

### DESCRIPTIVE STATISTICS

The descriptive statistics of the study variables are given in **Table 1**. As can be seen, the teachers in the current sample reported an average EE of *M* = 2.27 (*SD* = 0.66). Enjoyment is the most prominent emotion teachers reported experiencing while teaching in 99% of the lessons, at least to some extent (rated 2 or higher; *M* = 2.81, *SD* = 0.54), whereas they reported experiencing anxiety only to a very small extent (*M* = 1.09, *SD* = 0.49); however, anxiety cannot be neglected completely, as teachers indicated feeling anxious at least to some extent in 8% of the lessons (for similar results, see Frenzel et al., 2009). Teachers experienced anger at least to some extent in 39% of the lessons (*M*= 1.61, *SD* = 0.49). As indicated by the intra-class correlations [ICCs(1)], emotional experiences appear to be quite situation-specific: only about 20% of variance in emotions lies between teachers, with the largest amount of variance being within teachers, in other words, on the lesson-level.

**Table 1:** Descriptive statistics of study variables.

	*M*	*SD*	%^1^	ICC(1)
**Trait**
Emotional exhaustion	2.27	0.66	–	–
Emotional labor	2.33	0.80	–	–
**State**
Emotional labor	1.48	0.54	38/28^2^	0.39
Enjoyment	2.81	0.54	99	0.23
Anxiety	1.09	0.18	8	0.19
Anger	1.61	0.49	39	0.20

*All variables were assessed on a rating scale ranging from 1 to 5.*

*^1^Gives the percentage of lessons, where the respective construct is rated 2 or higher.*

*^2^The first value indicates the percentage of lessons for the suppression item, the second for the faking item.*

Teachers engage to a moderate extent in EL (trait: *M* = 2.33, *SD* = 0.80; state: *M* = 1.48, *SD* = 0.54). In the momentary assessments, altogether with the 794 responses, teachers indicate that they suppress their emotions more often (in 38% of the lessons) than they fake emotions (28%). When compared to emotional experiences, EL seems to be more person-specific; 39% of the variance is between teachers, yet the largest amount is still within teachers on the lesson-level.

Intercorrelations of all study variables are given in **Table 2**. On the between-level, EE is positively related to trait EL, but not to (aggregated) state EL (only having a marginally significant relationship, *p* < 0.10). Furthermore, the more exhausted teachers indicate they were, the lower their aggregated state enjoyment, and the higher the reported aggregated state anger will be; no significant relationship was found with aggregated state-anxiety. Aggregated state EL is significantly related to aggregated state-anxiety and anger, yet not to enjoyment. Emotions are not correlated significantly to each other on the between-level, meaning that teachers reporting having experienced enjoyment often do not also report less anxiety or anger. Regarding within-level relations, state emotions are correlated significantly to each other. Thus, in teaching situations where teachers report having experienced some enjoyment, they also report less anger and anxiety. Finally, EL is negatively related to enjoyment and positively related to anxiety and anger on the within-level.

**Table 2:** Intercorrelations of study variables.

	(1)	(2)	(3)	(4)	(5)	(6)
**Trait**
(1) Emotional exhaustion	1	0.51***	0.22	-0.44**	0.14	0.40**
(2) Emotional labor	-	1	0.48***	-0.44**	0.33	0.39**
**State**
(3) Emotional labor	-	-	1	-0.08	0.51*	0.90***
(4) Enjoyment	-	-	-0.35***	1	-0.04	-0.14
(5) Anxiety	-	-	0.24***	-0.18***	1	0.44
(6) Anger	-	-	0.48***	-0.42***	0.13*	1

The values below the diagonal give the intercorrelations on the within-level, above the diagonal for the between level.

*p < 0.05, **p < 0.01, ***p < 0.001.

### RELATING EMOTIONAL EXHAUSTION TO TEACHERS’ STATE EMOTIONAL EXPERIENCES

According to previous empirical evidence, teachers’ EE should be mirrored in the experience of diminished levels of positive and elevated levels of negative emotions while teaching. To address this, we regressed teachers’ state emotional experiences (enjoyment, anxiety, and anger) on their trait reported EE (Hypothesis 1; see **Table 3**). As hypothesized, EE relates negatively to teachers’ enjoyment (*b* = –0.35, *p* < 0.05) and positively to teacher anger (*b* = 0.25, *p* < 0.01). The effect sizes are moderate (*R*^2^_enjoyment/anger_ = 0.20/0.16). There was no significant relationship between EE and teachers’ experiences of anxiety.

**Table 3:** Predicting teachers’ state emotional experiences by trait reported emotional exhaustion.

	Enjoyment		Anxiety		Anger	
	Estimate	*SE*	Estimate	*SE*	Estimate	*SE*
Intercept (γ_00_)	2.76	0.08	1.09	0.03	1.61	0.07
Emotional exhaustion (γ_01_)	–0.35*	0.15	0.03	0.04	0.25**	0.08
*R*^2^	0.20		0.02		0.16	

Unstandardized coefficients are shown. Emotional exhaustion was entered as a grandmean-centered predictor into the respective model. The explained variance *R*^2^ refers to the explained variance on the between level.

*p < 0.05, **p < 0.01.

### RELATING EMOTIONAL EXPERIENCES AND EMOTIONAL EXHAUSTION TO STATE EMOTIONAL LABOR

In order to untangle the relations of teachers’ emotional experiences and EE and their state-reported EL (Hypotheses 2 and 3), we ran three successive regression models (see **Table 4**).

**Table 4:** Predicting teachers’ state emotional labor by emotional experiences, emotional exhaustion, and trait emotional labor.

	Emotional labor (state)					
	M1		M2		M3	
	Estimate	*SE*	Estimate	*SE*	Estimate	*SE*
**Within**
Enjoyment (γ_10_)	-0.10**	0.03			-0.11**	0.03
Anxiety (γ_20_)	0.30**	0.11			0.30**	0.11
Anger (γ_30_)	0.34***	0.05			0.34***	0.05
**Slope variance**
Enjoyment (Var *u*_1_*_j_*)	0.01	0.01			0.01	0.01
Anxiety (Var *u*_2_*_j_*)	0.06	0.06			0.03	0.03
Anger (Var *u*_3_*_j_*)	0.06***	0.01			0.03*	0.01
**Between**
Enjoyment (γ_01_)	0.02	0.09			0.07	0.07
Anxiety (γ_02_)	0.63	0.36			0.43	0.30
Anger (γ_03_)	0.88***	0.21			0.89***	0.20
Emotional exhaustion (γ_04_)			-0.02	0.10	-0.15	0.09
Emotional labor (trait) (γ_05_)			0.35**	0.12	0.22***	0.06
***R***^**2**^
Within	0.39		0.38
Between	0.67	0.24	0.73

Unstandardized coefficients are shown. Level 1-predictors are entered groupmean centered into the model. The respective emotions as predictors on level 2 were grandmean centered and introduced as the per-person aggregated emotions based on the level 1 data.

*p < 0.05, **p < 0.01, ***p < 0.001.

On the within-level (see M1), all emotions are predictive for EL as hypothesized, with enjoyment being negatively related to EL (*b* = –0.10, *p* < 0.01) and anxiety and anger positively related to EL (*b*_anxiety_ = 0.30, *p* < 0.01, *b*_anger_ = 0.34, *p* < 0.001). In turn, on the between-level, only anger is predictive of state EL (*b* = 0.88, *p* < 0.001). In total, 39 and 67% of variance in state EL are explained by emotional experiences on the within- and between-levels, respectively. Interestingly, the slope variance for anger is small, yet statistically significant, indicating that the relationship between momentarily experienced anger and EL differs between teachers.

The relationship between EE and state-reported EL is close to zero when controlling for trait EL (M2). Both trait variables explain 24% of variance in teachers’ state EL. When comparing explained variances on the between-level in M1, M2, and M3, it can be seen that 6% of explained variance is unique to trait EL and trait EE.

Introducing all predictors into the regression equation (M3) leaves the coefficients for emotional experiences fairly unchanged: on the within-level, all emotions are predictive of state EL; on the between-level, only anger and trait EL are statistically significant as predictors of state EL.

## DISCUSSION

The purpose of the study was to investigate teachers’ emotional processes, or their experiences of discrete emotions and EL, and relate it to EE. The ESM was employed to tackle these emotional processes on a state level and assess them during in-class instruction, thus allowing for intraindividual analyses. This is the first study to do so.

### THE ROLE OF EMOTIONAL EXHAUSTION FOR TEACHERS’ MOMENTARILY EXPERIENCED EMOTIONS

Regression analyses showed that teachers’ overall level of EE is indeed reflected in their emotional experiences while in class: the more exhausted teachers indicated they were, the less experiences of enjoyment and the more experiences of anger they indicated. The experience of anxiety was not related to EE. Only a handful of studies have directly examined this relationship, and our findings confirm the previously shown relationship between negative emotions and EE (Carson, 2007; Chang, 2013).

The majority of previous studies on teacher burnout do not examine teachers’ (discrete) emotions related to teaching in class as either consequences of or antecedents (or both) to EE as the core dimension of burnout. In one recent correlational study, Chang (2013) tested both directions of the relationship (burnout leads to negative emotions or negative emotions leads to burnout) and concluded that the intensity of negative emotions from one episode accounted for teacher burnout. Our study adds new understandings of such a relationship by confirming that EE (trait-level) could contribute to the experience of enjoyment and anger (state-level); we didnot test for the reverse effect (emotional experiences on EE) because EE was assessed prior to the ESM-period. Given the mixed findings of the directions, we believe future studies could continue unpacking this complex and dynamic relationship by employing a qualitative approach (by interviewing teachers, for example) coupled with a quantitative design that allows for determining causal links.

### THE RELATION OF TEACHERS’ MOMENTARILY EXPERIENCED EMOTIONS TO EMOTIONAL LABOR

Analyses revealed that teachers regularly suppress or fake their emotions. The teachers in our sample reported having engaged in EL and employing surface acting strategies (suppression or faking) to regulate their state emotions in about one third of the covered lessons. In addition, EL is significantly related to teachers’ experiences of anger on an inter-individual level. This is congruent with previous results from inter-individual analyses that indicated the prevalent relevance of anger within the context of EL and EE (Sutton and Wheatley, 2003; Chang and Davis, 2009). Teachers’ experience of anxiety is only related to EL on an intra-individual level, yet no such relation exists when comparing teachers.

Regarding teachers’ experience of enjoyment, a negative relationship between enjoyment as the desired emotional experience (as indicated by implicit display rules) and EL was anticipated. Intra-individually, this was supported by the study results: a teacher who experiences more enjoyment in a given teaching situation reports lower levels of EL. This conclusion cannot be drawn based on inter-individual results; here, the relation between enjoyment and EL is close to zero. One explanation for this finding may be the differences of interrelations between *emotions* on the intra- and inter-individual levels: whereas emotional experiences correspond to each other on a situational basis (state correlations; see **Table 2**), teachers who experience more enjoyment do not necessarily also report less anger (lack of inter-correlations on an inter-individual level). Thus, in a given teaching situation, the experience of enjoyment goes along with reduced levels of anxiety and anger, thus also reducing the necessity for engaging in EL. Lacking the inter-individual relationships between emotions, this explanation cannot hold true for the (lack of) inter-individual relationship between enjoyment and EL. Future studies could address this issue and investigate the relations between emotions as they are intra-individually experienced in situations, and to what extent these relations hold true for inter-individual differences. Also, future investigations could address the specific role enjoyment plays in EL and emotion regulation in general, by, for example, including other emotion regulation strategies than those considered within the present study.

Regarding the strength of the relationship between teachers’ momentarily experienced emotions and EL, there seem to be differences between teachers regarding the emotion of anger; in other words, how strongly anger relates to EL on a situational level differs between teachers. This could be indicative that, given a specific level of anger, some teachers regulate their anger expression to a larger extent than do other teachers. This might be due to teacher and/or situational differences in emotion regulation strategies not covered within the present investigation (e.g., deep acting; Brotheridge and Lee, 2003; Hochschild, 2012) and would warrant further investigations in the future. Given that EL is clearly associated with EE, identifying the factors that lead some teachers to deal with their anger in a given teaching situation more adaptively might be beneficial to prevent exhaustion.

### METHODOLOGICAL CONSIDERATIONS ON THE ASSESSMENT OF TEACHERS’ EMOTIONAL PROCESSES

To overcome several drawbacks from previous studies on teachers’ emotional lives, we employed the ESM (Carson et al., 2010; Keller et al., 2014) to assess teachers’ emotional experiences and EL momentarily while they were in class and teaching. In particular, trait emotions, that is, emotions as assessed on a generalized level, are assumed to be biased and do not necessarily reflect the actual emotions as experienced in the situation (see Robinson and Clore, 2002). Thus, the present assessment of teachers’ state emotions instead of commonly used trait emotions can overcome this methodological flaw and provide insight into teachers’ momentary emotional experiences in a highly ecologically valid way.

Related to this, we found EL as assessed during a concrete teaching situation to be only moderately related to EL as assessed on a generalized trait-level. The question arises regarding how reliably trait reports capture teachers’ emotion-related constructs as they actually occur in a given teaching situation. This issue should be addressed and explicitly investigated in future studies. We also found anxiety to be only of subordinate importance when it came to teachers’ emotional lives in class. Two reasons seem likely to explain this finding. First, the overall low values of state-anxiety could be due to employing the actual emotion-word for assessing the respective emotion. The word “anxiety” implies high arousal levels, which presumably occur very seldom during a lesson and would be captured by a random assessment even less often; yet, anxiety as an emotion covers also low-arousal states of anxiety, such as nervousness, which might not be covered by our assessment (compared with somewhat higher values for teachers’ state-anxiety using the item wording: “I was tense and nervous during this lesson” in Frenzel et al., 2009). Second, and related to the previously mentioned trait-state ambiguity, is the fact that trait emotions might reflect something other than the actual emotions experienced in a concrete situation. Thus, teachers’ trait-reported emotions differ in their magnitude from state-reported emotions, including anxiety (Keller et al., 2014). As such, the present findings of low anxiety levels while teaching might be a first indicator that anxiety occurs less frequently in the actual teaching situation, but more so in retrospect when evaluating situations over a longer time frame and employing personal beliefs when doing so.

Teachers spend the majority of their time teaching, and this can be considered the central task in which they engage (OECD, 2011); yet their emotional lives in the classroom have not been explicitly addressed to date. Thus, the ESM allows us to tap into teachers’ emotional lives during instruction and covers an important — perhaps the most important — aspect of teachers’ professional lives. While in class, complex interactions with students require teachers to constantly monitor and regulate their affective image, thereby drawing on self-regulatory resources. Thus, addressing emotions and EL while teaching is highly relevant.

Lastly, the ESM assessment as utilized in the present study allows for intra-individual analyses of teachers’ emotional processes. Unraveling intra-individual functioning is a core goal in personality psychology (Eid and Diener, 1999); yet, the majority of research on teachers’ emotions focuses on inter-individual differences. In the present study, we were able to separate differences between teachers from differences occurring across situations, yet within-teachers. As findings on the relationship between emotions and EL indicate, the results that were yielded on an intra-individual level are not necessarily transferable to the inter-individual level.

### LIMITATIONS

Due to the ESM design, the present investigation is subject to some limitations. First, all variables as implemented in the current study were assessed via self-reports. While that may be justified, as only the individuals themselves can report on their concrete subjective affective experiences and stress-related variables, other measures complementing self-reports, such as physiological measures of arousal, are called for and could be implemented in future studies.

The teacher sample in the present study is rather small. The ESM assessment yielded an adequate sample size on the within-level, however, multilevel analyses demand between 30 and 50 units on the between-level for reliably estimating between-level effects and differences (Maas and Hox, 2004). Thus, the present sample of 39 teachers should yield reliable results, yet a replication of study findings would be helpful. Also, the teachers participating in the present study all teach in one school track (the Gymnasium in Germany); future studies could also consider other school types to gain a more comprehensive picture.

Strictly speaking, our cross-sectional design does not allow us to model causal effects. In fact, regarding emotional processes in the context of teachers’ stress and EE, effects are most likely reciprocal. For example, given that EE leads to an increase in the experience of negative emotions, these in turn would necessitate an increase in EL efforts, thereby depleting resources that could ultimately lead to higher levels of exhaustion. Future studies could combine momentary assessment with a longitudinal study design to unravel both intra-individual and long-term processes and effects.

## CONCLUSION

The present study fills a gap in the existing literature by investigating emotional processes within the context of teachers’ EE (see Chang, 2009). It employs the ESM to assess teachers’ discrete momentary emotions and EL while teaching. This approach allows us to address intra-individual emotional processes and relate them to inter-individual differences in teachers’ EE.

In investigating emotional processes as they occur in a given teaching situation, the present study cannot draw any conclusions as to what causes teachers’ emotional reactions in class. Previous investigations have shown the paramount importance of students’ (mis)behavior in the context of teacher EL and exhaustion (Chang and Davis, 2009; Tsouloupas et al., 2010). Thus, students’ lack of adherence to stated classroom rules or obstruction of teachers’ goals could cause teachers to experience anger (Sutton and Wheatley, 2003; Sutton, 2007; Frenzel et al., 2009); the present investigation shows that experiences of anger necessitate teachers to engage in EL and are also a correlate of teachers’ EE. One may speculate that exhaustion leads to more frequent experiences of anger, or exhaustion is a consequence of increased experiences of anger, or both. Thus, findings of the present study imply the beneficial effects of anger reduction, which should lead to less EL, and possibly over a longer time frame, also to less exhaustion. Reducing one’s experience of anger might be viable by so-called reappraisal strategies (see for example Gross and John, 1998). Future studies could develop intervention programs for teachers based on reappraisal training for a reduction of anger experiences during class and investigate the effects of such training on EL and consequently, exhaustion or well-being in teachers.

Beyond the prevalent importance of anger, the present study’s results also indicate the potential of enjoyment in reducing teachers’ EL: on a situational level, increasing the experience of positive emotions might decrease teachers’ engagement in EL, thus reducing their risk of eventually suffering from exhaustion and burnout. Positive emotions have previously been suggested to act as an important resource (Fredrickson, 1998), yet empirical results backing that theoretical claim have been missing so far. Beyond the implication that enjoyment as an appropriate emotion (according to implicit display rules) demands less EL efforts, the negative relationship between enjoyment and EE on an intra-individual level points toward its importance as a possible resource in the teaching context. Thus, increasing teachers’ experiences of enjoyment could act as a buffer for teacher burnout.

Although our study hints at this possible relationship and its implications, further efforts are needed to explicitly investigate this link. Thus, future investigations should focus on emotional experiences of teachers and how they — causally — relate to EL and burnout on an intra-individual level, including the characteristics of the situation and how it is perceived and appraised by teachers (on the role of appraisals and coping strategies within the context of burnout, see for example, Chang, 2009, 2013). Including situation characteristics and how they are perceived and appraised by teachers would allow for identifying teaching situations (as characterized by student behavior, for example) and how they influence teachers’ emotional experiences, which would ultimately provide the means for interventions designed to shape beneficial emotional experiences that reduce teachers’ risk for burnout.

## Conflict of Interest Statement

The authors declare that the research was conducted in the absence of any commercial or financial relationships that could be construed as a potential conflict of interest.
